# Tacrolimus-induced neurotoxicity from bipolar disorder to status epilepticus under the therapeutic serum level: a case report

**DOI:** 10.1186/s12883-021-02479-z

**Published:** 2021-11-15

**Authors:** Bora Jin, Ga Yeon Kim, Sang-Myung Cheon

**Affiliations:** Department of Neurology, School of Medicine, Dong-A University, 26, Daesingongwon-ro, Seo-gu, Busan, 49201 Korea

**Keywords:** Tacrolimus, Neurotoxicity, Bipolar disorder, Ataxia, Status epilepticus

## Abstract

**Background:**

Tacrolimus is a macrolide immunosuppressant widely used to prevent rejection after solid organ transplantation. In general, adverse events of tacrolimus occur more often as the concentration of tacrolimus in the blood increases. We report the case of a 39-year-old man who developed a variety of adverse events despite in the therapeutic level of tacrolimus in the blood.

**Case presentation:**

A 39-year-old man underwent liver transplantation for liver cirrhosis due to alcoholic liver disease. The postoperative immunosuppressant consisted of tacrolimus (5 mg) and mycophenolate (500 mg) twice daily. Five months after taking tacrolimus, he presented with talkativeness, which gradually worsened. Brain magnetic resonance imaging performed 10 months after tacrolimus administration revealed a hyperintense lesion affecting the middle of the pontine tegmentum on T2WI. The blood concentration of tacrolimus was 7.2 ng/mL (therapeutic range 5–20 ng/mL). After 21 months, he exhibited postural tremor in both the hands. Twenty-four months after taking tacrolimus, he showed drowsy mentality, intention tremor, and dysdiadochokinesia. Electroencephalography presented generalized high-voltage rhythmic delta waves; therefore, tacrolimus was discontinued in suspicion of tacrolimus-induced neurotoxicity, and anticonvulsive treatment was started. The level of consciousness gradually improved, and the patient was able to walk independently with mild ataxia.

**Conclusion:**

This case shows that tacrolimus-induced neurotoxicity can occur even at normal concentrations. Therefore, if a patient taking tacrolimus exhibits psychiatric or neurologic symptoms, neurotoxicity should be considered even when the blood tacrolimus is within the therapeutic range.

## Background

Tacrolimus is a macrolide immunosuppressant. It blocks the production of interleukin 2, which inhibits T-lymphocyte proliferation [1]. The neurological side effects of calcineurin inhibitors (cyclosporine and tacrolimus) are most commonly mild, such as headache, dysarthria, visual changes, and postural tremor [2]. Severe side effects can include psychosis, cortical blindness, and seizures [2]. Severe complications have been more frequently reported following liver and lung transplantation than with renal transplantation, which typically occur with tacrolimus concentrations consistently above the therapeutic range of 15 ng/mL [3]. Neurotoxicity commonly occurs early after the initiation of tacrolimus; however, it may occur months or even years later [2]. Here, we report a case of tacrolimus-induced neurotoxicity despite in the therapeutic blood level of tacrolimus.

## Case presentation

**Fig. 1:**
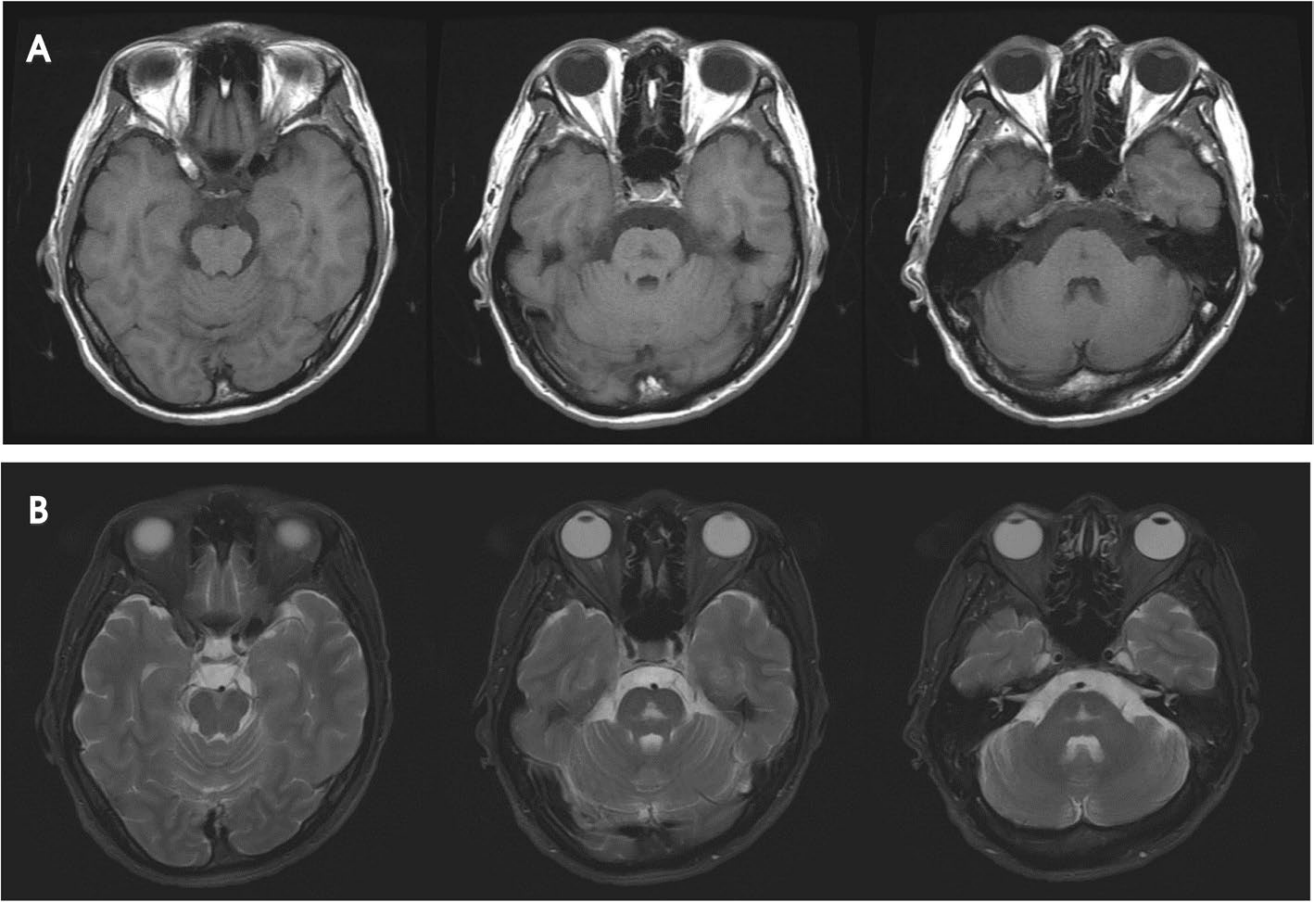
Brain MRI at 10 months after taking tacrolimus shows a low signal intensity lesion in the middle of the pontine tegmentum on a T1 weighted image (**A**) and high signal intensity lesion in the same region on a T2 weighted image (**B**)

**Fig. 2:**
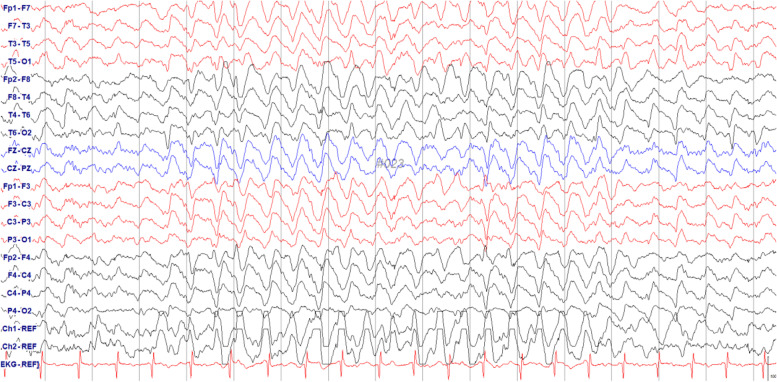
Electroencephalography at 24 months after taking tacrolimus shows generalized high-voltage rhythmic delta waves

**Fig. 3:**
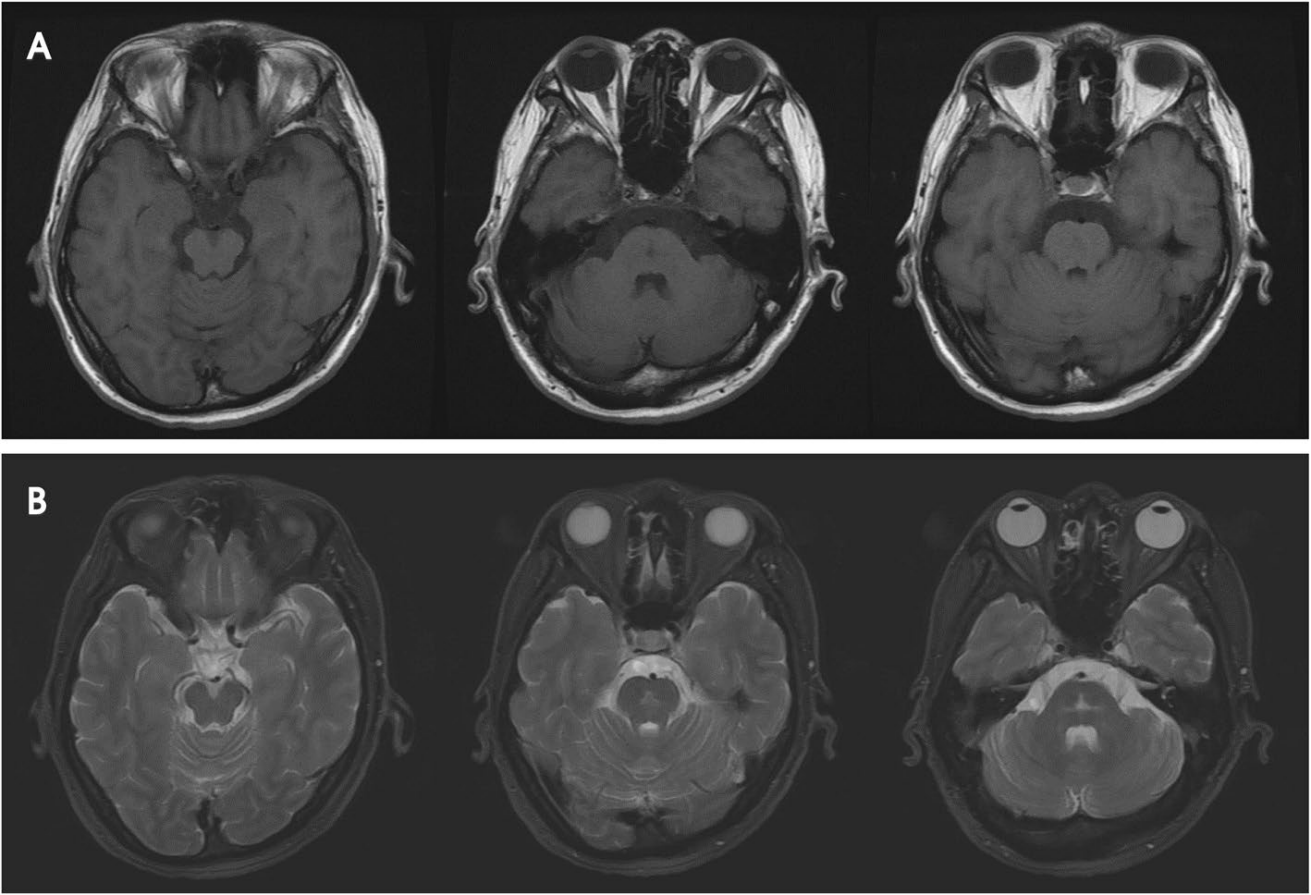
Brain MRI at 4 months after stopping tacrolimus shows partial resolution of low signal intensity lesion in the middle of pontine tegmentum on T1WI (**A**) and high signal intensity in the same region on T2WI (**B**)

**Fig. 4:**
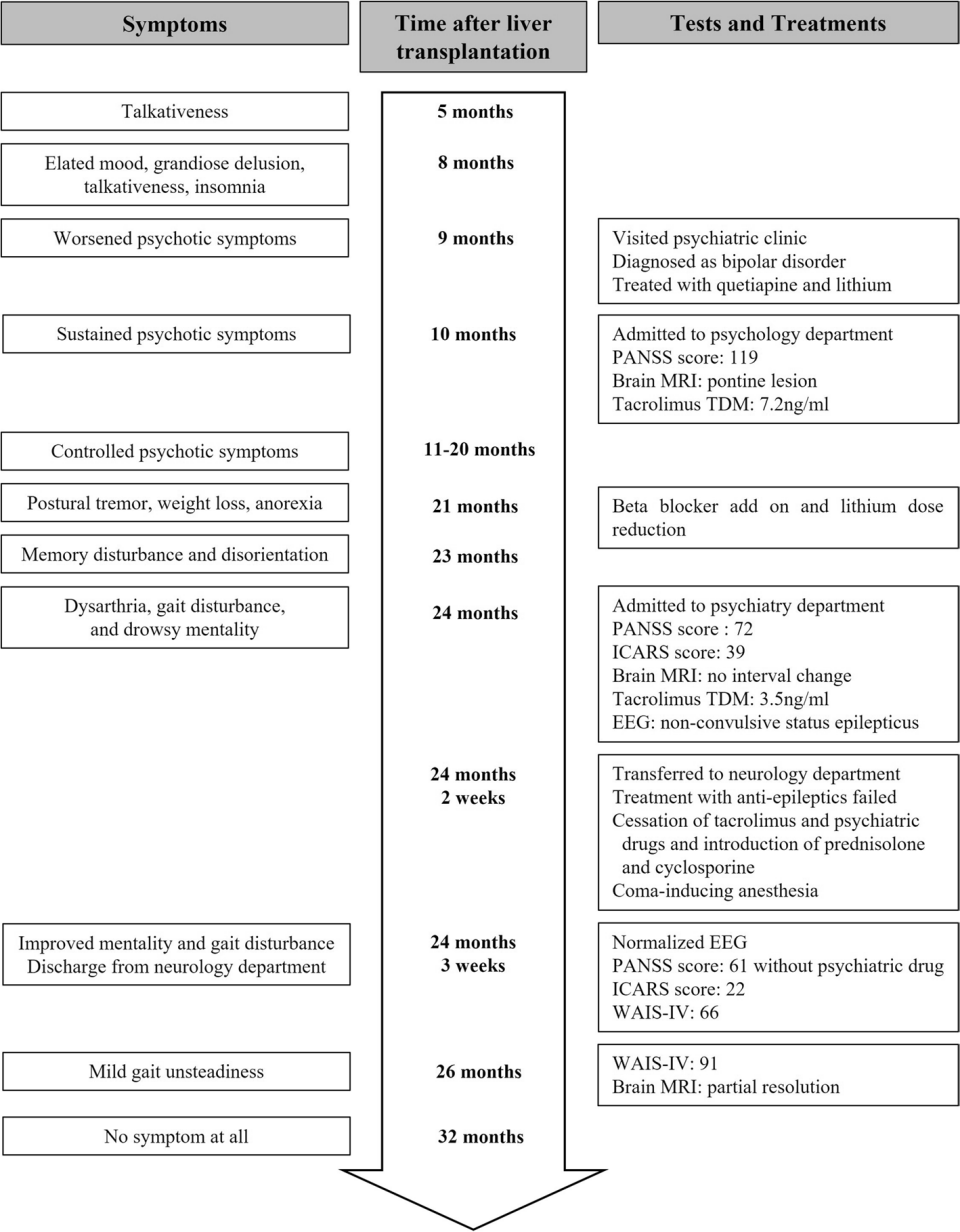
Time course of symptoms, signs, and treatments of the patient

A 39-year-old man underwent liver transplantation for liver cirrhosis due to alcoholic liver disease. He had no other medical history, including absence of any neuropsychiatric disorder. Postoperative immunosuppressive therapy consisted of tacrolimus (5 mg) and mycophenolate (500 mg) twice daily. Five months after taking tacrolimus, he presented with talkativeness, which gradually worsened. Three months later, he showed elated mood, grandiose delusion, talkativeness, and a decreased need for sleep, which was diagnosed of bipolar disorder. At that time, he was not taking any medication other than tacrolimus and mycophenolate mofetil. Short-term hospitalization and discharge were repeated due to poor control of these symptoms and lack of insights. Brain magnetic resonance imaging (MRI) performed 10 months after tacrolimus administration revealed a hyperintense lesion affecting the middle of the pontine tegmentum on T2 weighted images without enhancement (Fig. 1). Blood concentration of tacrolimus was 7.2 ng/mL (therapeutic range: 5–20 ng/mL). There were no laboratory abnormalities other than a slight elevation in AST levels, including electrolytes and vitamins. The score on the Positive and Negative Syndrome Scale (PANSS) for schizophrenia was 119. The introduction of antipsychotic drugs, such as lithium and quetiapine, controlled these symptoms for about a year. Twenty-one months later, he exhibited postural tremor in both hands, which was reduced with the administration of a beta-blocker. Two months later, he presented with memory disturbance and disorientation, which progressed with dysarthria and gait disturbance. He was hospitalized and referred for neurologic consultation. The score of PANSS score for schizophrenia was 72. On neurologic examination, he presented with drowsy mentality, kinetic tremor, intention tremor, dysdiadochokinesia, postural instability, and inability to stand in tandem. The International Cooperative Ataxia Rating Scale score was 39 [4]. The concentration of tacrolimus was 3.5 ng/mL. Brain MRI showed the same lesion in the pontine tegmentum. No laboratory abnormalities except for the presence of albumin-cytologic dissociation protein (64 mg/dL) in the cerebrospinal fluid were detected. No anti-neuronal or paraneoplastic autoantibodies were detected. During the examination, the patient’s level of consciousness rapidly deteriorated, and electroencephalography (EEG) showed generalized high-voltage rhythmic delta waves, which were terminated by intravenous injection of lorazepam (Fig. 2). Non-convulsive status epilepticus was diagnosed, and anticonvulsant treatment was initiated. Administration of tacrolimus was stopped in suspicion of tacrolimus-induced neurotoxicity, and the immunosuppressive regimen was changed to mycophenolate and prednisolone. Despite anticonvulsant treatment and discontinuation of tacrolimus, the ictal rhythm persisted and was eventually controlled through coma-inducing anesthesia with mechanical ventilation. Thiopental was administered, and the dose was gradually increased to achieve burst suppression in EEG, which was made 32 h later and maintained for an additional 24 h. The patient’s level of consciousness gradually improved over 2 weeks of aggressive treatment and produced normal EEG. Immunosuppressive treatment was maintained with cyclosporine instead of prednisolone. The patient was able to walk independently with mild ataxia (ataxia score 22) and showed a schizophrenia score of 61 without any psychiatric medication. The Wechsler Adult Intelligence Scale-IV score was 66, corresponding to intellectual disability at the time of discharge after 3 weeks of hospitalization. The patient was administered cyclosporine as an immunosuppressant without any other medication. Two months after discharge, the follow-up intelligence score was 91, corresponding to average level of intelligence. The follow-up brain MRI showed partial resolution of the lesion in the middle of the pons (Fig. 3). The patient did not present any neurological or psychiatric sign or symptom 8 months after discharge (Fig. 4).

## Discussion and conclusion

The cellular basis of neurotoxicity associated with tacrolimus has not been conclusively identified [5]. Tacrolimus mediates immune-suppressive effects via inhibition of calcineurin, which provokes a complete block off in the translocation of the cytosolic component of the nuclear factor in activated T-cells, resulting in a failure to activate the genes regulated by the nuclear factor of activated T-cells transcription factor [5]. Tacrolimus decreases the expression of p-glycoprotein as a drug efflux pump in brain endothelial cells and causes dysfunction of the blood-brain barrier, resulting in vasogenic edema [5]. In this patient, neurologic manifestation could be considered as a result of mycophenolate mofetil; however, such cases have rarely been reported and are irrelevant to the patient’s presentation. Neurotoxicity is a well-known adverse event associated with tacrolimus-mediated immunosuppression after solid organ transplantation. Approximately, 25–31% of patients who receive tacrolimus experience some form of neurotoxic adverse events [6]. They have mild neurotoxicity, including tremors (most common), insomnia, headache, dysesthesia, and mood disturbance. These adverse events generally occurred within the first 30 days following transplantation and were correlated in many instances to high plasma levels of tacrolimus [6]. Sakamoto et al. investigated the correlation between neurotoxicity and intracerebral concentration of tacrolimus in rats and demonstrated that at concentrations over a threshold value (700 ng/g of the rat brain), the intensity of the neurological event increases with the concentration of tacrolimus in the brain [7]. The recommended target concentration of tacrolimus in combination with mycophenolate is 6–10 ng/mL during the first 4 weeks following transplantation and 5–8 ng/mL thereafter [8]. A neurological event can occur at therapeutic blood concentrations [9], and even within the therapeutic range there could be a possibility of increased permeability of the blood brain barrier or decreased elimination from the brain in those patients. However, there are no reports on sudden onset of various complications appearing at once, as in our case. Of those, serious neurotoxicity such as medication-resistant status epilepticus can occur and suggests the importance of EEG for altered mentality. Prompt recognition of tacrolimus-induced neurotoxicity may be challenging. Our patient developed psychiatric symptoms 5 months after taking tacrolimus, which progressed until the brain MRI showed abnormal lesions in the pons. Such lesions could have resulted from other causes, such as myelinolysis due to hyponatremia or vitamin deficiency; however, no such events occurred during the patient’s illness. Despite these clues, the diagnosis was delayed by 2 years. Therefore, if a patient taking tacrolimus exhibits psychiatric or neurologic symptoms, neurotoxicity should be considered even within the therapeutic range of blood tacrolimus.

## Data Availability

Not applicable.

## Abbreviations

PANSS: Positive and Negative Syndrome Scale
EEG: Electroencephalography
MRI: Magnetic resonance imaging
